# Assessing Coral Reefs on a Pacific-Wide Scale Using the Microbialization Score

**DOI:** 10.1371/journal.pone.0043233

**Published:** 2012-09-07

**Authors:** Tracey McDole, James Nulton, Katie L. Barott, Ben Felts, Carol Hand, Mark Hatay, Hochul Lee, Marc O. Nadon, Bahador Nosrat, Peter Salamon, Barbara Bailey, Stuart A. Sandin, Bernardo Vargas-Angel, Merry Youle, Brian J. Zgliczynski, Russell E. Brainard, Forest Rohwer

**Affiliations:** 1 Biology Department, San Diego State University, San Diego, California, United States of America; 2 Department of Math and Computer Sciences, San Diego State University, San Diego, California, United States of America; 3 Pacific Islands Fisheries Science Center, NOAA Fisheries, Honolulu, Hawaii, United States of America; 4 Scripps Institution of Oceanography, University of California San Diego, La Jolla, California, United States of America; 5 Rosenstiel School of Marine and Atmospheric Science, University of Miami, Miami, Florida, United States of America; 6 Joint Institute for Marine and Atmospheric Research, University of Hawai'i at Manoa, Honolulu, Hawaii, United States of America; Leibniz Center for Tropical Marine Ecology, Germany

## Abstract

The majority of the world's coral reefs are in various stages of decline. While a suite of disturbances (overfishing, eutrophication, and global climate change) have been identified, the mechanism(s) of reef system decline remain elusive. Increased microbial and viral loading with higher percentages of opportunistic and specific microbial pathogens have been identified as potentially unifying features of coral reefs in decline. Due to their relative size and high per cell activity, a small change in microbial biomass may signal a large reallocation of available energy in an ecosystem; that is the *microbialization* of the coral reef. Our hypothesis was that human activities alter the energy budget of the reef system, specifically by altering the allocation of metabolic energy between microbes and macrobes. To determine if this is occurring on a regional scale, we calculated the basal metabolic rates for the fish and microbial communities at 99 sites on twenty-nine coral islands throughout the Pacific Ocean using previously established scaling relationships. From these metabolic rate predictions, we derived a new metric for assessing and comparing reef health called the microbialization score. The microbialization score represents the percentage of the combined fish and microbial predicted metabolic rate that is microbial. Our results demonstrate a strong positive correlation between reef microbialization scores and human impact. In contrast, microbialization scores did not significantly correlate with ocean net primary production, local chl*a* concentrations, or the combined metabolic rate of the fish and microbial communities. These findings support the hypothesis that human activities are shifting energy to the microbes, at the expense of the macrobes. Regardless of oceanographic context, the microbialization score is a powerful metric for assessing the level of human impact a reef system is experiencing.

## Introduction

The relationship between increasing human activity and decreasing fish biomass is well-established in coral reef systems [1]–[3]. Although herbivore reduction due to overfishing probably facilitates coral to algal transitions, the mechanistic link between overfishing and coral mortality is not clear [4]. Much uncertainty about the mechanisms of reef decline linked to eutrophication and climate change also still exists [5]–[6]. In addition to increasing algal cover relative to hard coral cover, other effects of anthropogenically-driven disturbances include disease outbreaks, fewer links in trophic webs, and loss of physical structure and habitat complexity [7]–[9]. Reef-associated microbial communities have been shown to respond to all of the above disturbances (overfishing, nutrient enrichment, thermal stress) by becoming less beneficial and more pathogenic, i.e. the proportion of sequences related to known pathogens typically increases [10]–[17].

Despite the epidemiological evidence linking the microbial ecology of coral reef systems to human activity, the largest study of coral reef microbial communities included only four coral atolls in the Line Islands, all clustered within one oceanographic region [14]. In this island chain a 10-fold increase in microbial and viral abundances in the overlying reef-water correlated with increasing human disturbance and was accompanied by decreased fish biomass [1], [14]. Further, a large proportion of the microbial 16S rDNA sequence similarities on the most disturbed reefs were most closely related to known pathogens [14]. These reefs also had the highest incidences of coral disease and the lowest percent coral cover. Other studies have also suggested that the total carbon flow through microbial pathways via detritus is inversely related to coral cover [18]–[19].

Ecosystems exhibit higher-level properties resulting from lower-level phenomena [20]. The energy available to a higher trophic level, for example, is reduced by the amount required to support the individual organisms in the lower level. The Metabolic Theory of Ecology (MTE) predicts the metabolic rate of individual organisms based on the observation that most variation in an individual's metabolic rate can be explained by body size and temperature [21], [22]. Whole organism metabolic rate (*I*), defined as the amount of energy per unit time that an individual organism requires, is calculated using Equation 1:
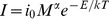
(1)Where 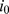 is the mass-independent normalization constant, 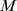 is the wet weight of the organism in grams, and 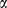 is the scaling exponent. The effects of temperature on metabolic rate are accounted for by 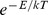 [21], [23] where *E* is the activation energy, *k* is Boltzmann's constant (8.62×10^−5^ eV K^−1^), and *T* is the water temperature at the site at the time of collection (in Kelvin). Distinct scaling exponents have been derived for different physiological states and evolutionary groups [21], [24]–[25].

The process of replacing macroorganisms with microbes has been termed *microbialization* [26]. In this study, Equation 1 was used to predict metabolic rates for all individual fish and microbes present in a 10 m^3^ volume of reef water. *Microbialization* refers to an increase in the percentage of the combined fish and microbial predicted metabolic rate that is microbial. Island-level microbialization scores were derived for 29 islands (99 sites) within four oceanographic regions of the Pacific Ocean. Our data show a strong significant positive correlation between microbialization scores and the NCEAS cumulative human impact scores at each island. In comparison, microbialization scores did not correlate with the net primary production values. These findings support the hypothesis that human activities rather than variation in oceanographic conditions are causing microbialization of coral reefs and that the microbialization score is a powerful metric for assessing and comparing reef health.

## Materials and Methods

### Site descriptions

**Figure 1:**
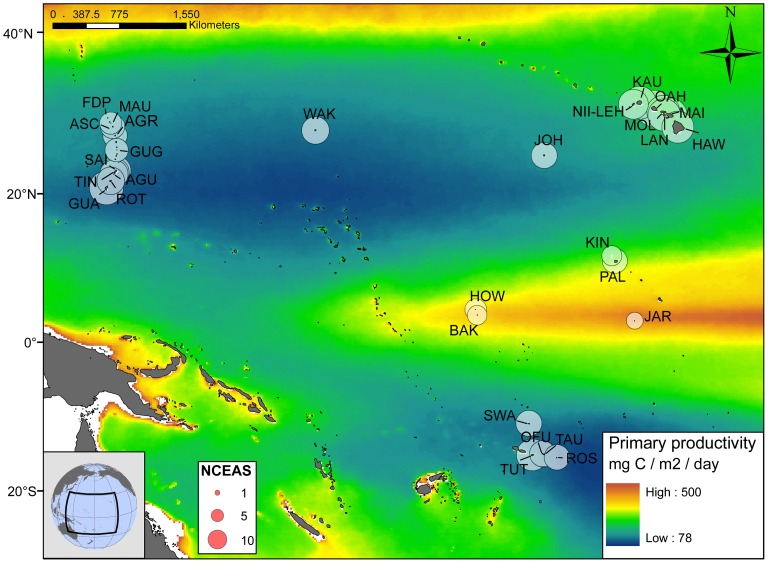
Location of the 29 islands surveyed. Color scale indicates oceanic net primary production derived from satellite data using the Vertically Generalized Production Model (VGPM). Circles indicate the relative NCEAS cumulative human impact score for each island. For island abbreviations see Table 1.

The twenty-nine islands included in this study were surveyed following the National Oceanic and Atmospheric Association (NOAA) 's Rapid Ecological Assessment (REA) protocol as part of the Coral Reef Ecosystem Division (CRED) and Pacific Reef Assessment and Monitoring Program (Pacific RAMP) [27]. Multiple coral reef sites (average depth: 10 m) were sampled at each island in four broad regional groups: the Main Hawaiian Islands (MHI), Guam and the Mariana Islands (MARIANA), the American Samoa region (SAMOA), and the Pacific Remote Island Areas (PRIA) (Fig. 1, Table 1). Microbial samples were collected during the 2008–2010 Pacific RAMP monitoring cruises: MHI (2008), MARIANAS (2009), SAMOA (2010), PRIA (2010). For fish, belt survey data from 2001–2009 was used for all islands. Because the REA survey protocol switched to the Stationary Point Count (SPC) method in 2009, 2010 fish data was not included. Microbial and fish data collection sites at each island are not necessarily co-located. Due to the variability inherent with observational fish data, the standard approach for estimating island means for fish abundance requires a large sample size. To have an adequate sample size, this fish data was pooled from all sites and years. Island-level averages and standard errors for fish and microbial biomass are provided in Table S2 and Fig. S2. Microbial metabolic rates were calculated per site then averaged by island. Island-level averages for fish and microbial predicted metabolic rates were used to calculate one microbialization score for each island.

**Table 1:** Survey data and calculated values for 29 islands in the Pacific, grouped by region.

REGION		MICROBIAL COMMUNITY			FISH COMMUNITY		OTHER		
Code	Island	Abundance x 10^5^	Total Biomass	Predicted Metabolic Rate	Total Biomass	Predicted Metabolic Rate	NPP	Chla	NCEAS Score
		cells ml^−1^	g 10 m^−3^	W 10 m^−3^	g 10 m^−3^	W 10 m^−3^	mg C m^−2 ^yr^−1^	µg l^−1^	
**GUAM & MARIANA ISLANDS (orange)**									
AGR	Agrihan	2.6	0.22	0.005	84.54	0.007	155	0.11	7.7
AGU	Aguijan	2.3	0.16	0.006	41.5	0.005	125	0.34	9.9
ASC	Asuncion	2.7	0.15	0.002	182.54	0.011	159	0.11	7.6
FDP	Farallon de Pajaros	2.7	0.2	0.003	103.18	0.007	165	0.06	6.8
GUA	Guam	2.8	0.27	0.012	17.98	0.002	126	0.17	13.7
GUG	Guguan	3.5	0.27	0.002	145.03	0.012	153	0.1	7.1
MAU	Maug	3	0.24	0.003	70.95	0.005	159	0.22	6.7
ROT	Rota	2.3	0.17	0.003	36.9	0.004	125	0.07	9.4
SAI	Saipan	2.1	0.21	0.017	23.31	0.003	143	0.1	11.2
TIN	Tinian	1.8	0.17	0.004	31.19	0.003	143	0.05	10.3
**MAIN HAWAIIAN ISLANDS (MHI, blue)**									
HAW	Hawaii	4.7	0.81	0.012	51.24	0.004	248	0.12	12.2
KAU	Kauai	2.8	0.69	0.024	33.39	0.002	262	0.34	13
LAN	Lanai	3.3	0.4	0.007	33.44	0.002	264	0.15	12.7
MAI	Maui	3	0.56	0.019	40.16	0.003	258	0.21	14.2
MOL	Molokai	2.1	0.32	0.006	24.8	0.002	270	0.1	12.8
NII/LEH	Niihau & Lehua	4.1	1.29	0.05	54.49	0.003	234	0.22	10.7
OAH	Oahu	3.7	1.53	0.076	23.99	0.002	270	0.19	15.6
**PACIFIC REMOTE ISLANDS & ATOLLS (PRIA, pink)**									
BAK	Baker	3.8	0.33	0.004	228.18	0.011	380	0.1	5.3
HOW	Howland	4.5	0.49	0.014	195.37	0.022	380	0.06	6.3
JAR	Jarvis	5.8	0.46	0.005	408.75	0.026	445	0.08	4
JOH	Johnston	3.5	0.72	0.024	91.6	0.005	196	0.09	8.5
KIN	Kingman	1.7	0.18	0.002	514.84	0.015	282	0.11	5.5
PAL	Palmyra	3.7	0.22	0.002	229.08	0.01	307	0.16	8
WAK	Wake	2.2	0.12	0.001	161.4	0.008	147	0.06	9.5
**SAMOA REGION (green)**									
OFU/OLO	Ofu & Olosega	2.9	0.19	0.003	57.83	0.004	139	0.07	8.4
ROS	Rose	3.2	0.14	0.002	82.98	0.007	130	0.04	8.2
SWA	Swains	3.1	0.26	0.004	85.17	0.005	148	0.04	8.6
TAU	Tau	3.3	0.23	0.004	44.77	0.004	139	0.06	8.6
TUT	Tutuila	3.5	0.25	0.006	33.11	0.003	151	0.15	12.4

Predicted metabolic rates are basal rates. NPP = net primary production. Colors identify each island group in the figures.

### Collection of microbial data

At each site, 4 replicate 2 l seawater samples were collected ∼1 m above the benthos using polycarbonate Niskin bottles. Microscopy grade glutaraldehyde was added to a final concentration of 0.3% v/v. Microbial cells were collected from each sample by filtration using a 0.2 µm Anodisc filter (Whatman) and then stained with 5 µg ml^−1^ DAPI (Molecular Probes, Invitrogen) within 2 hours of collection [28]–[30]. Filters were mounted on microscope slides and stored at −20°C. For each site, 10 fields of view (5 fields for each of 2 replicate filters, ∼200 cells per field) were examined by epifluorescence microscopy (excitation/emission: 358/461 nm) at 600× magnification. Cell counts and dimensions were collected using ImagePro Software (Media Cybernetics) set for a size range of 0.00001–10 µm for both length and width. Cell volume (*V*) was calculated by considering all cells to be cylinders with hemispherical caps using Equation 2:
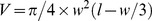
(2)where *l* is length and *w* is width [31]. No correction was made for possible cell shrinkage as a result of fixation. Individual microbial cell volumes 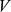
(µm^3^) were converted to mass in wet weight (g) using previously established size-dependent relationships for marine microbial communities [32]. Each cell volume *V* was next converted to dry weight using the linear relationship derived from data reported in Simon and Azam (1989) and shown in Equation 3:
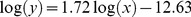
(3)where *x* is cell dry weight and *y* is cell volume (r^2^ = 0.99). Then cell wet weight (*z*) was calculated using the linear relation shown in Equation 4 [32] (r^2 = ^0.99):
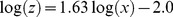
(4)


### Collection of fish data

This study includes fish data from all surveys performed at REA sites during the years 2001–2009. The number of REA sites surveyed over this time period is provided for each island in Table S2. Visual surveys provided a census of the reef fish community [33]. Surveys were restricted to shallow-to-moderate depths along the forereef between 10–15 m with a majority of surveys completed along the 10 m isobaths. At each site, a total of three 25 m long belt transect surveys were conducted by two different divers. For each survey, the diver made two passes: during the first pass, all fish >20 cm in length were recorded in adjacent 4 m wide belts; during the second pass all fish ≤20 cm were recorded in 2 m wide belts. Lengths were recorded to the nearest cm for fish <5 cm and in 5 cm bins for all others [34]. Species-specific mass values for individual fish were calculated from length-weight relationships using FishBase [35]–[36]. The fish data was provided for each family as mean biomass (g m^−2^) and mean abundance (# individuals m^−2^), from which the mean mass per individual (g) was calculated. Because surveys were carried out at an average water depth of 10 m and surveyors counted all fish in the water column up to the surface, the mean abundances (individuals per m^2^) represented the total number present in a 10 m^3^ water column.

### Metabolic rate calculations

At each REA site, community-level metabolic rates were calculated by summing the individual metabolic rates (*I*) for all fish or microbes present in a standard volume of water (10 m^3^). Individual metabolic rates (*I*) in watts were calculated using Equation 1.

The mass independent normalization constant for fish, i_0_, (ln[i_0_] = 18.47) was extracted from the plots in Brown et al. [21], while those for basal and active microbial states (4.61×10^16^ and 1.08×10^21^, respectively) were calculated from previously reported individual prokaryote metabolic rate values [25]. The predicted scaling exponents (α) used for microbes were 1.72 (basal) and 1.96 (active) [25], while 0.71 was used for fish [21]. The activation energies (*E*) used were 0.61 eV for microbes [25] and 0.69 eV for fish [21].

### Quantification of human impact

The level of human impact was assessed from the cumulative global human impact map generated by the National Center for Ecological Analysis and Synthesis (NCEAS; http://www.nceas.ucsb.edu/globalmarine/impacts). Using ArcGIS 9.3, “NoData” pixels corresponding to the land mass of each island were identified and converted into polygon format. A 10 km zone was then calculated for each of these polygons, representing the immediate 10 km of sea surface around the border of each island in the study. Using these zones, statistics were then performed on the NCEAS human impact raster in order to calculate the mean impact score. These scores incorporate data related to: artisanal fishing; demersal destructive fishing; demersal non-destructive, high-bycatch fishing; demersal non-destructive low-bycatch fishing; inorganic pollution; invasive species; nutrient input; ocean acidification; benthic structures (e.g., oil rigs); organic pollution; pelagic high-bycatch fishing; pelagic low-bycatch fishing; population pressure; commercial activity (e.g., shipping); and anomalies in sea surface temperature and ultraviolet insolation.

Other indicators of reef system health were also considered in this study using benthic survey data collected at the same time as the microbial data. Benthic surveys were performed using the survey methodology described in Vargas-Angel [37]–[38]. A principal components analysis was carried out using R on the following initial variables: coral disease prevalence, prevalence of coral colonies with compromised health (unidentified sub-lethal lesions including algal and cyanophyte interactions, and barnacle and tubeworm infestations), percent crustose corraline algae cover, percent coral cover, and the microbialization score [39], [40]. Raw data sets were rescaled to give mean 0 and standard deviation of 1. As a supplement to PCA analysis, *k*-means clustering was also performed on the same data matrix for *k* = 2–8 (100 iterations); the dissimilarity matrix was calculated using Gower's standardization [41].

### Estimation of net primary production

Productivity estimations for net primary productivity (NPP) (mg C m^−2^ day^−1^) were derived from Moderate Resolution Imaging Spectroradiometer (MODIS) satellite data using the Vertically Generalized Production Model (VGPM; http://www.science.oregonstate.edu/ocean.productivity/standard.product.php). This model, based on an algorithm by Behrenfeld and Falkowski (1997) calculates net primary production from satellite-based measurements of surface chl*a* concentrations, while also taking into account sea surface temperature, daily photosynthetically active radiation, and a temperature-dependent photosynthetic efficiency factor [42]. Because these satellite data sets are less accurate for near-shore measurements, the satellite-based NPP values used here were estimated from the data for a 50 km radius ring surrounding each island, with the first 10 km around each island removed. The nearshore chl*a* concentrations (µg l^−1^) used in this study were obtained using fluorometric analysis [43]. The chl*a* samples were collected in conjunction with the microbial samples at each site.

## Results and Discussion

### Predicted metabolic rates for the fish and microbes

Field surveys carried out at 99 coral reef sites at 29 Pacific islands (Fig. 1) were used to calculate the biomass (g per 10 m^3^) and basal metabolic rate (W per 10 m^3^) for both the water column-associated microbial and fish communities (Table 1). The high and low values for microbial biomass occurred on the islands of Oahu (1.53 g per 10 m^3^) and Wake Atoll (0.12 g per 10 m^3^), respectively. This difference in microbial biomass equates to a 76-fold increase in the rate of energy flux (W per 10 m^3^ or J sec^−1^ 10 m^−3^) on Oahu (0.076 W per 10 m^3^) relative to Wake Atoll (0.001 W per 10 m^3^). The highest fish biomass was found on Kingman (514.84 g per 10 m^3^) and the lowest on Guam (17.98 g per 10 m^3^). The metabolic requirements predicted for the fish communities on Kingman and Guam were 0.015 and 0.002 W per 10 m^3^, respectively. This difference equates to a 7.5-fold reduction in the metabolic requirements of the fish community. The largest differences in the predicted metabolic rates between each island represent a 100-fold change for the microbes, as compared to a 14-fold change for the fish (Table 1).

### Microbialization scores versus the NCEAS human impact score

Based on the predicted metabolic rates for fish and microbes (Table 1), we are proposing a separate metric called the *microbialization score*, which represents the microbial share of the total predicted metabolic rate. The microbialization score is the percentage of the combined fish and microbial predicted metabolic rate that is microbial:
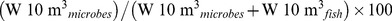
(5)


Although both increased microbial biomass and decreased fish biomass affect microbialization scores, microbial biomass has a proportionately greater impact on the combined predicted metabolic rate. For example, on Oahu, the fish are responsible for only 3% of the combined predicted metabolic rate, but account for 94% of the total biomass. Even on Kingman where we observed the highest fish biomass and microbial biomass represented less than 0.03% of the total biomass, the microbes still account for 13% of the combined predicted metabolic rate.

**Figure 2:**
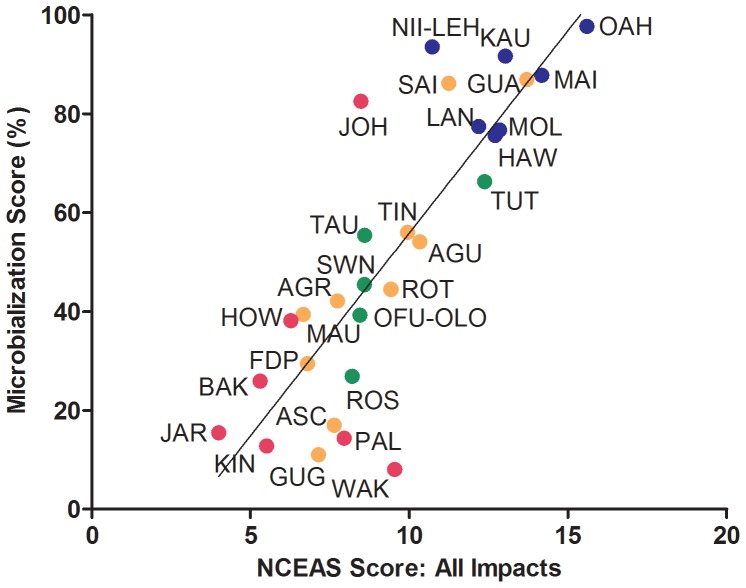
Linear regression analysis of microbialization scores versus NCEAS cumulative human impact values (y = 8.19 x – 26.10; r^2^ = 0.68). The microbialization score is the percentage of the combined fish and microbial predicted metabolic rate that is microbial. Color denotes oceanographic region: Guam and the Mariana Islands (orange circles), the Main Hawaiian Islands (blue circles), Pacific Remote Islands and Atolls (pink circles), and the Samoa region (green circles). For island abbreviations see Table 1.

Recently, the NCEAS human impact score has been proposed as a cumulative metric of different anthropogenic stressors ranging from overfishing to predicted climate change events [44]. As shown in Fig. 2, the microbialization score is positively correlated with the NCEAS score (linear regression, r^2 = ^0.68; Fig. 2). The microbialization scores ranged from 8% at remote and relatively pristine Wake Island to 75–98% in the heavily-impacted main Hawaiian Islands (MHI). Oahu, with the highest microbialization score (98%) also had the highest NCEAS score (15.59).

Johnston Atoll in the PRIA group appears to be an exception to the overall trend in that it has a high microbialization score (82%) but a relatively low NCEAS score (8.48). In actuality, Johnston is heavily impacted by factors not reflected in the NCEAS scores including the addition of two artificial islands with paved runways formed by coral dredging, usage for both above-ground and underground nuclear tests in the 1950s and 1960s, and service as a chemical weapons depot until 2000. The microbialization score appears to be a better indicator of these stressors than the NCEAS index of human impact.

A principal components analysis was carried out with the goal of visualizing how the microbialization score related to other indicators of reef health, including coral disease prevalence, prevalence of coral colonies with other signs of compromised health, percent crustose corraline algae cover, and percent coral cover (Fig. S1). The first two components accounted for 66% of the variation. The first component (PC1) accounted for 46% of the variation and was driven in the positive direction (relative to 0) by coral disease incidence, other visible signs of compromised coral health, and microbialization score (Fig. S1). A complete table of PCA loadings is provided in Table S3. By comparison, variables which typically correlate positively with reef system health (% crustose coralline algal cover and % coral cover) were represented as vectors moving in the negative direction (relative to 0). The separation by vector sign along PC1 supports the hypothesis that the microbialization score is a useful measure of reef system decline. Because the PCA analysis indicated that there was separation in the data, we used k-means clustering as a supplementary analysis to determine how many groups there were. K-means is a classical variance-based clustering method that defines *n* data points in *d* dimensions, into *k* clusters, so that the within clusters sun-of-squares is minimized [41]. The within group sum of squares plotted against the number of clusters (*k*) indicated *k = *3 to be the optimal number (for *k* = 2–8). The 11 islands contained in cluster two (within-cluster sum of squares = 1.47) were negative for PC1 (Fig. S1), while the 16 islands contained in cluster 3 were all positive on PC1 (within-cluster sum of squares = 2.06). The two islands in the first *k*-means cluster were Lanai (LAN) and Guam (GUA) (within cluster sum-of-squares = 0.32).

### Microbialization scores versus combined metabolic rate

**Figure 3:**
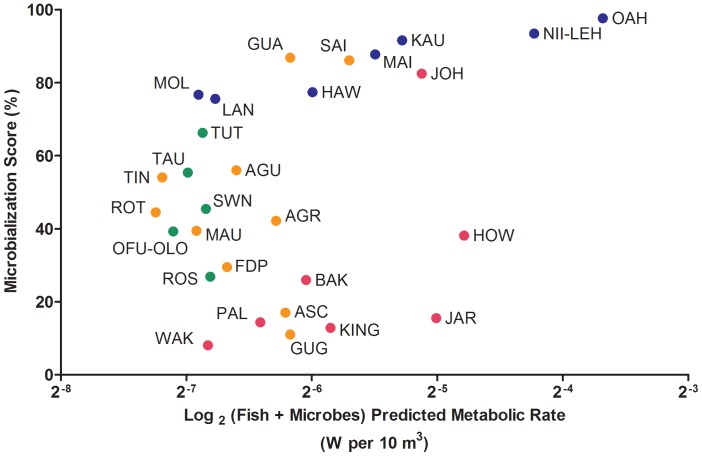
Microbialization scores plotted against the combined fish + microbes predicted metabolic rates for each of the 29 islands surveyed. Colors are as in Fig. 2. For island abbreviations see Table 1.

The metabolic rates predicted for the combined microbial and fish communities at the 29 islands ranged by approximately one order of magnitude, from a low of 0.007 W per 10 m^3^ on Rota Island (MARIANA) to a high of 0.077 W per 10 m^3^ on Oahu (MHI) (Fig. 3, x-axis). The combined predicted metabolic rate was not correlated with the microbialization score, which also varied widely among the islands, ranging from a low of 8% at Wake to a high of 97% at Oahu (Fig. 3, y-axis). At the low end of this scale, increased microbalization scores were explained by reduced metabolic contribution from the fish. However, higher microbialization scores were associated with a sharp rise in combined predicted metabolic rate driven primarily by increasing microbial metabolic rates. This break-point may reflect the release of the microbes from some resource limitation.

### Predicted metabolic rates of fish and microbes versus primary production

Net primary production (NPP) might be expected to be a significant factor driving variation in community metabolic rates. Previous small-scale inter-island studies that correlated differences in microbial communities with varying local human impacts could not conclusively rule out inter-island variations in oceanographic conditions as a possible driving factor [14]. To address this issue, we surveyed net primary production (NPP) at islands in four oceanographic regions throughout the Pacific Basin (Table 1).

**Figure 4:**
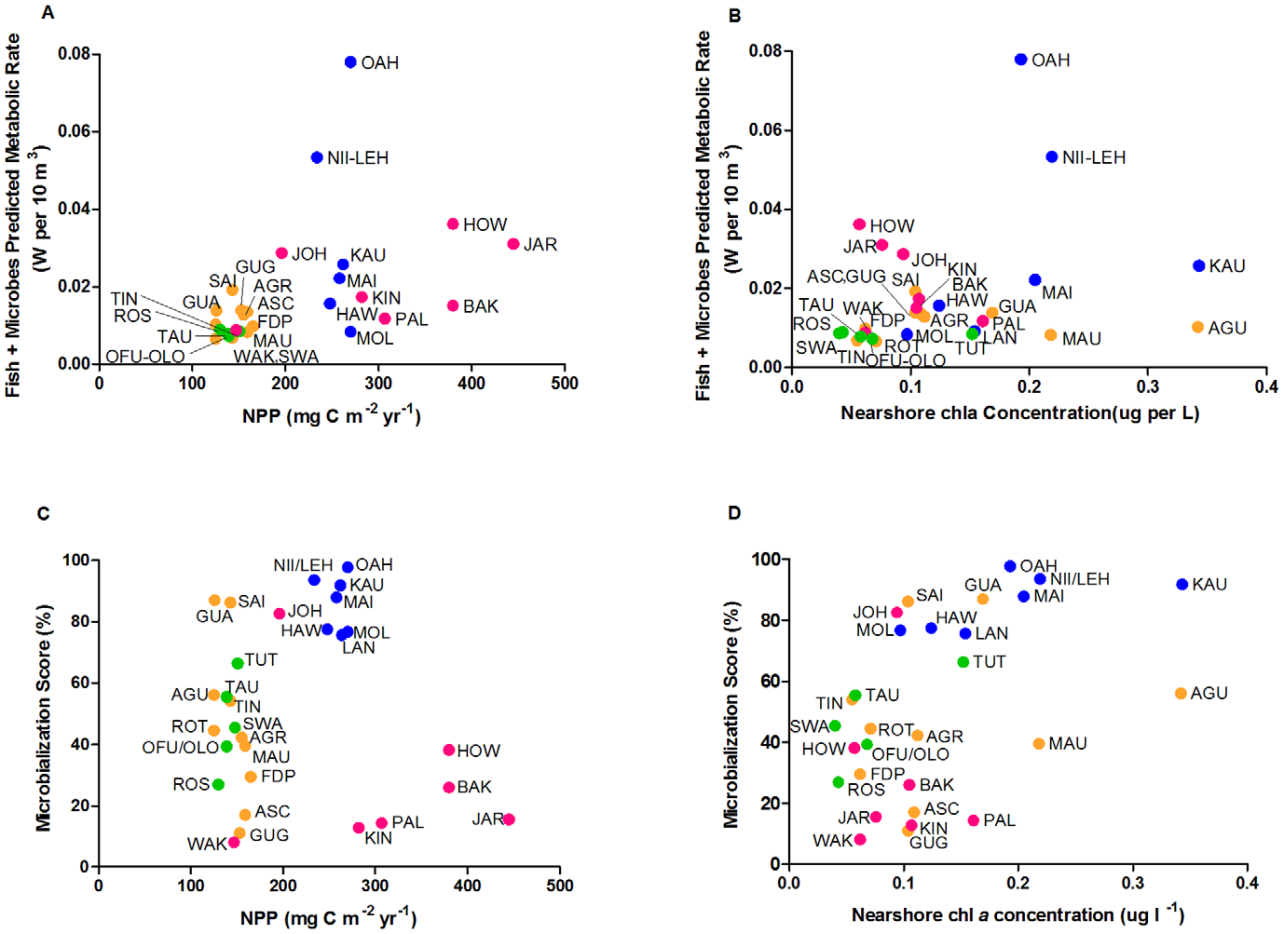
Measures of energy use versus metrics of primary production. (**a**) Non-linear regression analysis of the combined fish + microbes predicted metabolic rate versus net primary production (NPP) for the 29 surveyed islands. NPP was derived from satellite data using the Vertically Generalized Production Model (VGPM). (y = 0.00008x+0.0012; R^2^ = 0.21) (**b**) Non-linear regression analysis of the combined fish + microbes predicted metabolic rate versus nearshore chl*a* concentrations at the 29 surveyed islands (y = 0.54x+0.01; R^2^ = 0.08) (**c**) Microbialization scores versus NPP derived from satellite data using the VGPM for the 29 surveyed islands. (**d**) Microbialization scores versus nearshore chl*a* concentrations at the 29 surveyed islands (y = 171.5x+29.7; R^2^ = 0.22). Colors are as in Fig. 2. For island abbreviations see Table 1.

Estimated net primary production (NPP; mg C m^−2^ day^−1^) derived from satellite data is shown in Fig. 1. NPP ranged from 125 mg C m^−2^ day^−1^ at Aguijan to 445 mg C m^−2^ day^−1^ at Jarvis (Table 1). This predicted NPP was not a strong predictor of the combined fish + microbial metabolic rate at the island-level (non-linear regression, R^2 = ^0.21; Fig. 4A). Likewise, when the predicted NPP values were compared against the metabolic rates of the fish and microbial communities separately, R^2^ values were 0.20 for fish and 0.054 for microbes (Table 1). Large differences in NPP were observed between the geographic regions surveyed, but relatively little variation within each one (Fig. 4A, C). Since the satellite data used for the above predictions omitted a 10 km ring around each island, nearshore chl*a* concentrations were also measured as an alternative proxy for NPP. These samples were collected with the microbial samples at each site. The nearshore chl*a* concentrations (µg l^−1^) explained even less of the inter-island variation in combined predicted metabolic rate (non-linear regression, R^2 = ^0.08; Fig. 4B). For the individual communities, R^2^ values were 0.13 and 0.15 for fish and microbes, respectively (Table 1).

### Microbialization scores and primary production

The island microbialization scores did not correlate with predicted oceanic NPP values (Fig. 4C, R^2 = ^0.004) or nearshore chl*a* concentrations (Fig. 4D; R^2 = ^0.22). However, higher nearshore chl*a* concentrations associated with microbialization scores above a certain threshold (∼70%) are suggestive of eutrophication processes linked to human impact (Fig. 4D) [45]. These analyses demonstrate that estimated reef primary production is not a significant driver of variation in either community metabolic rates or microbialization scores.

To further examine whether or not accounting for oceanographic context would improve our ability to predict reef microbialization, multiple linear regression analysis was performed. In addition to the NCEAS score, satellite-based estimates of net primary production (NPP) and nearshore [chl*a*] were included as variables. This resulted in 4 models of interest: microbialization score = β_0_+β_1_(NCEAS)_,_ y = β_0_+β_1_(NCEAS)+β_2_(chl*a*), y = β_0_+β_1_(NCEAS)+β_2_(NPP), y = β_0_+β_1_(NCEAS)+β_2_(chl*a*)+β_3_(NPP). Given that the NCEAS score was in the model, the p-values for chl*a* and NPP were not significant by the t-test in the second and third models (p-value>0.1). The only variable that was significant was the NCEAS score, having a highly significant p-value in all of the models (p-value<0.0001). The model which included both chl*a* and NPP as variables (y = β_0_+β_1_(NCEAS)+β_2_(chl*a*)+β_3_(NPP)) gave a multiple R^2^ value of 0.706, which was not a significant improvement over the simplest model (y = β_0_+β_1_(NCEAS)) which explained 68.4% of the variability of the microbialization score.

Next, Akaike's Information Criterion (AIC) was used for model selection between the 4 different statistical models. AIC is the most widely known and used model selection criterion which consists of a “goodness-of-fit” term and a “penalty” term for increased number of model parameters [46]. The model with the lowest AIC value is selected as the best model. The model having the smallest AIC was the model which did not include additional variables (y = β_0_+β_1_(NCEAS)). Although the exact mechanism(s) underlying the *process* of microbialization remain unclear, these analyses support the hypothesis that human activities alter the energy budget of the reef system, specifically by altering the allocation of metabolic energy between microbes and macrobes.

The finding that microbialization scores did not significantly correlate with ocean net primary production, local chl*a* concentrations, or the combined metabolic rate of the fish and microbial communities suggests that the microbialization score may be a powerful metric for comparing and assessing reef degredation, particularly at large spatial scales. Other measures of reef degredation which are more heavily influenced by oceanographic context (i.e. percent coral cover, percent algal cover) may be more easily confounded by non-human factors and are subsequently harder to interpret across large spatial scales.

### Other considerations

In this study, surveys of microbial and fish sizes were used to predict whole organism metabolic rates. Ideally, the energetic requirements per unit time for fish and microbial communities would be measured empirically. However, this is not practical over this large region. To evaluate whether or not the MTE-based approach is a reasonable alternative to quantifying energy flux, the mean predicted metabolic rates for microbial communities were compared against experimental values reported from other studies (Table S1). The means for both the predicted basal metabolic rates used in our analyses and the corresponding predicted active metabolic rates fall within the same range as the empirically-based measurements.

Similarly, differences in temperature at the time of sampling explained a small proportion of the variation in metabolic rate between islands. Water temperature at the time of sampling ranged from 25–30°C. For the microbial community-level metabolic rates, the standard deviation in the 29 island data set was 0.16 at the actual temperatures and 0.01 when all locations were corrected to the same temperature (20°C); for the fish community-level metabolic rates, the standard deviations were 0.006 and 0.003, respectively. Temperature correction increased the r^2^ value for the regression analysis of community-level metabolic rate as a function of biomass by only 0.01% and 0.05% for fish and microbes, respectively. Therefore, inter-island variation in temperature does not account for our observed trends.

## Conclusions

Overfishing, eutrophication, and global climate change are important drivers of the global loss of coral reefs. However, the precise mechanism(s) by which these perturbations lead to coral decline have remained elusive. We and others have previously argued that human activities are favoring the coral reef-associated microbes at the expense of the macrobes, a process called *microbialization*. The data presented here supports this hypothesis over a wide swath of Pacific coral reefs and suggests that microbialization is a general process of reef decline. Although the exact mechanism(s) driving the process of microbialization remain unclear, the microbialization score provides a way to diagnose the degree of microbialization that has occurred on reefs. Fish were the primary movers of energy in the most pristine locations (i.e. fish were responsible for 97 and 87% of the total predicted metabolic rate on Wake (PRIA) and Kingman (PRIA), respectively) but made up only 3% of the total predicted metabolic rate on Oahu (MHI). Microbialization scores reflect both increased microbial biomass and decreased fish biomass; however microbial biomass has a proportionately greater impact on metabolic rate. This means that even a minor increase in the microbial load results in a substantial shift in community energy use; up to a 100-fold increase in the metabolic requirements of the microbes in the most heavily impacted reef systems. This study has significant implications for the protection of coral reefs. The degree of microbialization a reef is experiencing may be important for predicting its response to perturbation. On Pacific coral reefs, *microbialization* may be set in motion by an increase in the percent cover of turf algae resulting from the loss of herbivorous fish. Turf algae release large amounts of dissolved organic carbon (DOC) into the water column, a source of energy almost exclusively available to the microbes [47]. Consequently, the process of microbialization is likely to have stabilization effects in the system once a castastophic regime shift to an algal-dominated state has occurred.

## Supporting Information

Figure S1 **Principal components analysis of reef system properties related to reef health.** The first two principal components account for 66% of the variability in the dataset (PC1 = 46%, PC2 = 20%). Arrow length reflects the relative contribution of a variable to a PC axis. MS = microbialization score; CCA = % crustose coralline algae cover; DZ = % coral disease prevalence; CO = % coral cover; CH = % coral with other indications of compromised health. Symbol denotes oceanographic region: Guam and the Mariana Islands (*), the Main Hawaiian Islands (∧), Pacific Remote Islands and Atolls (#), and the Samoa region (+). Two groups of islands identified from k-means cluster analysis are divided along PC1 by the dotted line; the third group is circled (Lanai and Guam). For island abbreviations, see Table 1.
(TIF)

Figure S2 **Mean microbial and fish biomass.** (a) Mean microbial biomass with standard error. Total number of sites where microbial data was collected = 99. (**b**) Mean fish biomass with standard error. Total number of sites where fish data was collected = 791. The number of REA sites included is given in parentheses next to three-letter island code.
(TIF)

Table S1 **Comparison of mean MTE-based microbial metabolic rate predictions from this study with experimental measurements from marine systems^1–5^.** Rates of photosynthesis were converted from units of gross carbon production (P_g_) to units of power (W) using 39,444 J g^−1^ C, the standard free energy change from the synthesis of glucose from CO_2_ and H_2_O during photosynthesis at STP^6^. For conversion between rates of oxygen consumption or production in volume or mass units, we assumed that 1 ml O_2_ per second = 1.43 mg O_2_ per second^24^. To convert between units of power (W) and rates of respiration we assumed that 1W = 0.05 ml O_2_ per second^24^. Metabolic rates in W per 10 m^3^ were derived after calculating total daily energy use: P_g_ (from sunrise to sunset) + Respiration (over a 24 hour period). In studies where only dark incubation experiments were performed, total daily energy use was calculated assuming P_g_/R_24 hrs_ = 1. When two measurements are listed for the same sample and conditions, they indicate high and low values. B = predicted basal metabolic rate; A = predicted active metabolic rate.
(XLSX)

Table S2 **Summary table showing the number of REA sites where microbial or fish data (belt transect method only) was collected, time period of sampling, and standard error for biomass and abundance of the fish and microbial communities at each island.**
(XLS)

Table S3 **Summary table for Figure S1.** The importance of each component and the contribution (loadings) of each variable is shown. MS = microbialization score; CCA = % crustose coralline algae cover; DZ = % coral disease prevalence; CO = % coral cover; CH = % coral with other indications of compromised health.
(XLS)
