# Supplementary figures and images for: Assessing Coral Reefs on a Pacific-Wide Scale Using the Microbialization Score
Source: PLoS One. 2012 Sep 7;7(9):e43233. doi: 10.1371/journal.pone.0043233 (PMC3436891; doi:10.1371/journal.pone.0043233)

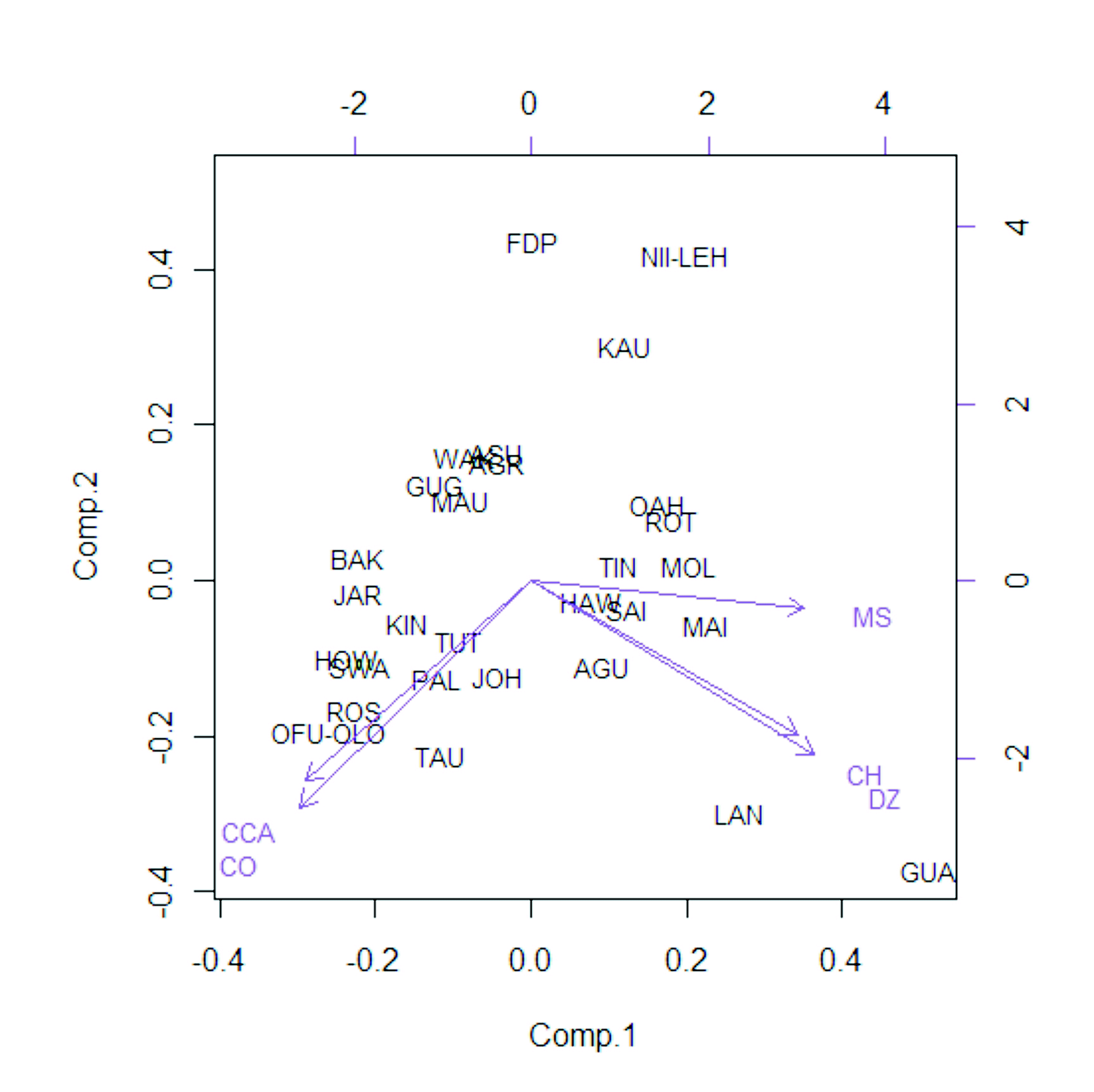

Supplement: Figure S1 — Principal components analysis of reef system properties related to reef health. The first two principal components account for 66% of the variability in the dataset (PC1 = 46%, PC2 = 20%). Arrow length reflects the relative contribution of a variable to a PC axis. MS = microbialization score; CCA = % crustose coralline algae cover; DZ = % coral disease prevalence; CO = % coral cover; CH = % coral with other indications of compromised health. Symbol denotes oceanographic region: Guam and the Mariana Islands (*), the Main Hawaiian Islands (∧), Pacific Remote Islands and Atolls (#), and the Samoa region (+). Two groups of islands identified from k-means cluster analysis are divided along PC1 by the dotted line; the third group is circled (Lanai and Guam). For island abbreviations, see Table 1. (TIF) [file pone.0043233.s001.tif]

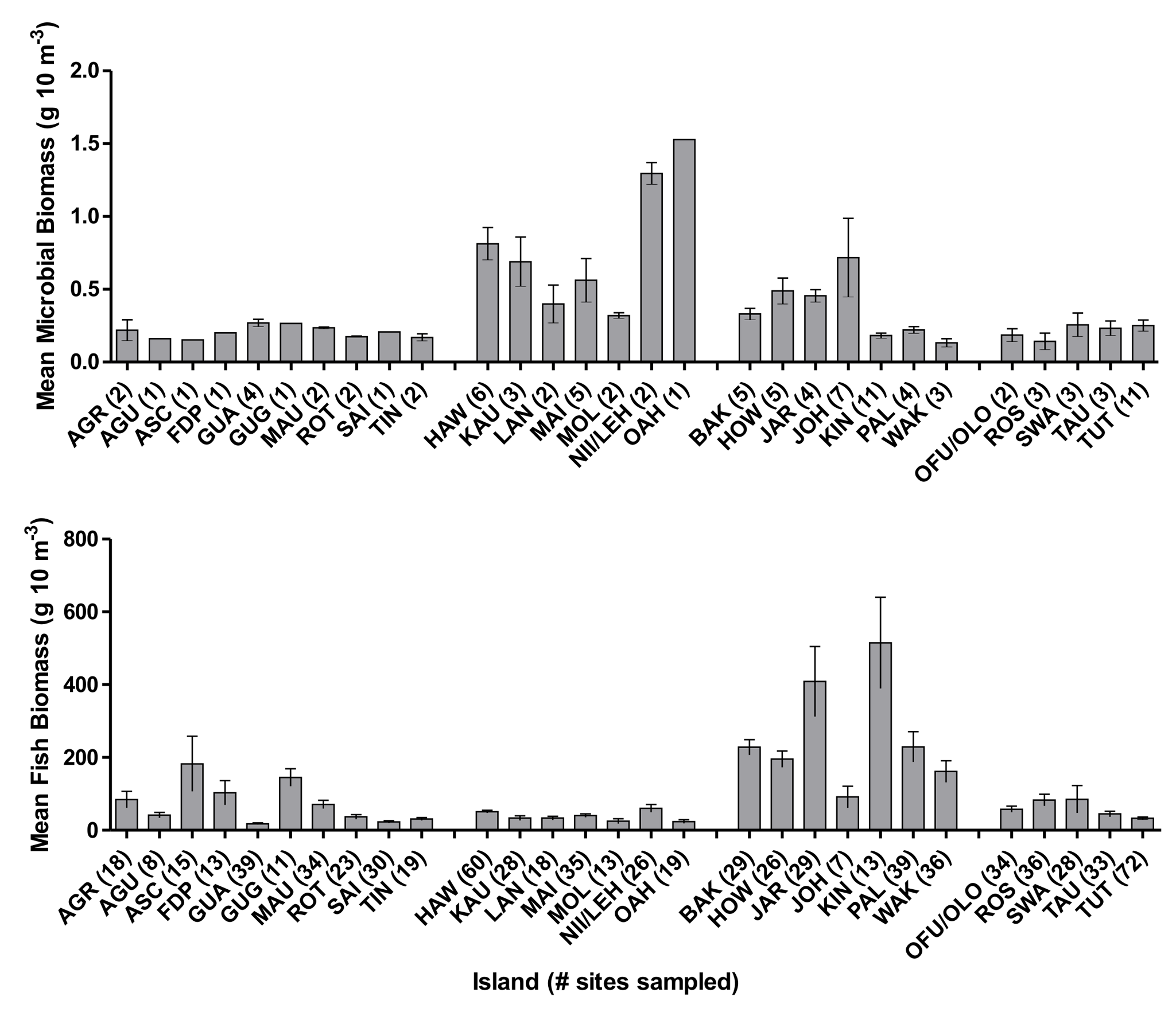

Supplement: Figure S2 — Mean microbial and fish biomass. (a) Mean microbial biomass with standard error. Total number of sites where microbial data was collected = 99. (b) Mean fish biomass with standard error. Total number of sites where fish data was collected = 791. The number of REA sites included is given in parentheses next to three-letter island code. (TIF) [file pone.0043233.s002.tif]
